# Influence of Ventilation on Formation and Growth of 1–20 nm Particles via Ozone–Human Chemistry

**DOI:** 10.1021/acs.est.3c08466

**Published:** 2024-02-07

**Authors:** Shen Yang, Tatjana Müller, Nijing Wang, Gabriel Bekö, Meixia Zhang, Marouane Merizak, Pawel Wargocki, Jonathan Williams, Dusan Licina

**Affiliations:** † Human-Oriented Built Environment Lab, School of Architecture, Civil and Environmental Engineering, École Polytechnique Fédérale de Lausanne (EPFL), 1015 Lausanne, Switzerland; ‡ Max Planck Institute for Chemistry, Hahn-Meitner Weg 1, 55128 Mainz, Germany; § International Centre for Indoor Environment and Energy, Department of Environmental and Resource Engineering, Technical University of Denmark, 2800 Kongens Lyngby, Denmark; ∥ School of Mechanical Engineering, Beijing Institute of Technology, 100081 Beijing, China; ⊥ Energy, Environment and Water Research Center, The Cyprus Institute, 2121 Nicosia, Cyprus

**Keywords:** indoor particles, air change rate, fan operation, VOCs, human skin, indoor chemistry

## Abstract

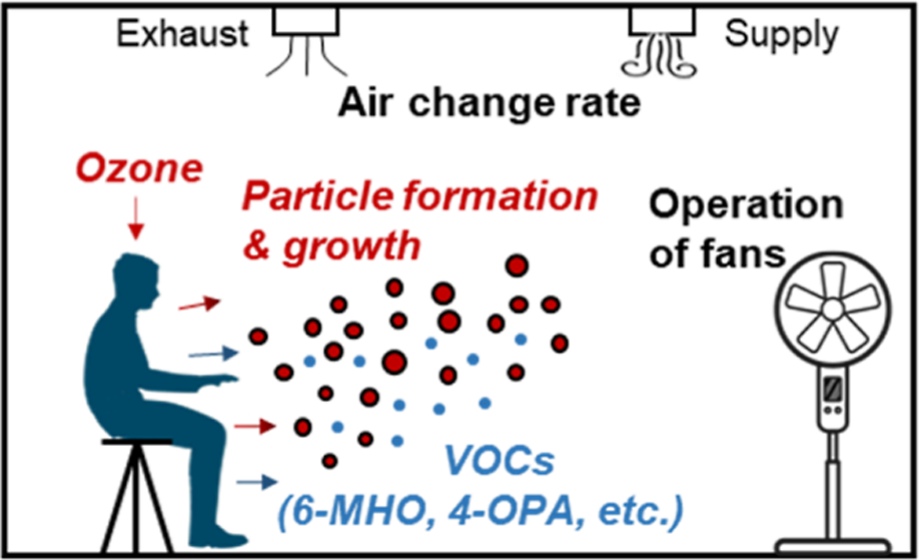

Ozone reaction with human surfaces is an important source of ultrafine particles indoors. However, 1–20 nm particles generated from ozone–human chemistry, which mark the first step of particle formation and growth, remain understudied. Ventilation and indoor air movement could have important implications for these processes. Therefore, in a controlled-climate chamber, we measured ultrafine particles initiated from ozone–human chemistry and their dependence on the air change rate (ACR, 0.5, 1.5, and 3 h^–1^) and operation of mixing fans (on and off). Concurrently, we measured volatile organic compounds (VOCs) and explored the correlation between particles and gas-phase products. At 25–30 ppb ozone levels, humans generated 0.2–7.7 × 10^12^ of 1–3 nm, 0–7.2 × 10^12^ of 3–10 nm, and 0–1.3 × 10^12^ of 10–20 nm particles per person per hour depending on the ACR and mixing fan operation. Size-dependent particle growth and formation rates increased with higher ACR. The operation of mixing fans suppressed the particle formation and growth, owing to enhanced surface deposition of the newly formed particles and their precursors. Correlation analyses revealed complex interactions between the particles and VOCs initiated by ozone–human chemistry. The results imply that ventilation and indoor air movement may have a more significant influence on particle dynamics and fate relative to indoor chemistry.

## Abstract 2

Ozone reaction with human surfaces is a source of ultrafine particles whose dynamics and fate can be significantly influenced by air change rate and air movement.

## 1 Introduction

Ultrafine particles are airborne particles with an aerodynamic diameter smaller than 100 nm (PM_0.1_). Originating directly from traffic emissions^1−4^ and via in situ atmospheric oxidation processes,^5,6^ ultrafine particles dominate the number distribution of urban aerosols.^7^ Exposure to ultrafine particles has been associated with respiratory and cardiovascular mortality and brain diseases.^8−10^ In indoor environments, where people spend most of their time,^11^ ultrafine particle levels can be comparable, or even higher than outdoor, owing to various indoor particle generation sources.^12−16^ Typical indoor ultrafine particle sources can be classified into three overlapping categories:^9^ combustion, such as cooking^17,18^ and burning candles;^19,20^ volatilization/nucleation/condensation, including those from electrical appliances,^21−23^ heated surfaces,^24^ and painting;^25^ and oxidation, mainly referring to ozone–terpene chemistry indoors.^26^ Ultrafine particle emissions have been documented from the ozone reaction with indoor terpene-rich fragrances,^27,28^ personal care products,^29−31^ and cleaning agents.^32,33^ A potentially strong yet understudied source of indoor ultrafine particles is ozone–human chemistry.

Humans play an important role in indoor ozone chemistry.^34^ Their skin lipids are rich in squalene and unsaturated fatty acids that can rapidly react with ozone.^35−37^ Gas-phase products from these reactions increase indoor levels of carbonyls, dicarbonyls, and hydroxycarbonyls.^38−43^ Additionally, human presence has shown impacts on secondary organic aerosols (SOAs) derived from ozone–limonene chemistry.^44^ A positive correlation between ozone and indoor ultrafine particle levels has been found in an occupied office.^45^ Laboratory experiments investigating ozone reactions with skin-oiled clothing^46^ and surface-sorbed squalene^47,48^ also reported noticeable ultrafine particle formations. However, these studies focused on >10 nm ultrafine particles (>6 nm in Fadeyi et al.^44^) and thus neglected the sub-5 nm size range in which the first step of new SOA formation takes place.^26^ Our previous study was the first to report nanocluster aerosol (NCA, sub-3 nm particles) formation via ozone reaction with humans.^49^ Yet, indoor particle growth beyond the NCA range remains understudied, and the impact of building ventilation and room air movement has not been considered.

Ventilation has direct impact on the outdoor-to-indoor transport of ozone and ultrafine particles, as well as dilution, residence time, and deposition of indoor reactants and products, thereby influencing indoor particle dynamics.^50−52^ Under a given ozone condition, lower ventilation rates are generally associated with higher levels of ultrafine particles generated from ozone chemistry.^53^ Rai et al. found that >10 nm particles formed via ozone reaction with skin-oiled clothing at an air change rate (ACR) of 0.5 h^–1^ but not when ACR increased to 2.7 h^–1^, although the ozone level remained similar.^46^ In ozone–human chemistry, ventilation rate has marginal effects on surface reactions but more pronounced impacts on gas-phase reactions,^37,54^ both being potentially related to particle formation and growth.^6,49^ In addition, fans are often used in chamber studies to force air mixing to homogeneity so that measurements at a single location can represent the overall level of particle concentrations inside the chamber.^55−57^ Occupants also utilize fans to improve thermal comfort in buildings.^58,59^ However, owing to the increased particle deposition at higher air velocity,^60^ it is expected that fan operation can impact particle formation and growth from ozone–human chemistry.

In summary, the formation and growth of 1–20 nm particles generated from ozone–human chemistry and their dependence on building operational conditions have not been sufficiently studied to date. Considering this knowledge gap, the objective of this study is to investigate the impact of ventilation, including both ACR and forced air mixing by indoor fans, on the formation and growth of 1–20 nm particles via ozone–human chemistry. In a controlled climate chamber, we quantified human-derived emissions of ultrafine particles and their dependence on ACR (0.5, 1.5, and 3 h^–1^) and mixing fan operation (on at all ACRs and off at 3 h^–1^). We also measured volatile organic compounds (VOCs) inside the chamber to explore the correlation between particles and gases resulting from ozone reactions on humans. The results of this study contribute to deepening our understanding of indoor SOA dynamics related to occupants and can be used for improving the prediction of occupants’ exposure to ultrafine particles and better ventilation control strategies to mitigate exposure to ozone-chemistry products.

## 2 Materials and Methods

### 2.1 Climate Chamber

We conducted a series of experiments within a 62 m^3^ climate-controlled chamber at the École Polytechnique Fédérale de Lausanne (EPFL), as shown in Figure S1. The chamber wall was made of stainless steel, and the ceiling was covered by aluminum foil, whereas the floor was covered by vinyl plates. The chamber’s ventilation system relied on 100% outdoor air, which underwent filtration using an F7 particle filter and a newly installed high-efficiency particulate absorbing filter in conjunction with an activated carbon molecular filter. This filtered air was then distributed through a supply diffuser and subsequently exhausted through a ceiling-mounted outlet. The climate within the chamber was tightly regulated by a dedicated heating, ventilation, and air-conditioning system, maintaining an air temperature of 24 ± 0.5 °C and a relative humidity of 50 ± 5% in the volume of the chamber. The chamber was furnished with three tables and six chairs. Two pedestal fans were placed in the chamber corners to investigate the influence of forced air mixing on particle formation by ozone–human chemistry. When the fans were on, they operated at the highest airflow rate at 1350 m^3^/h, at a fixed orientation aimed at the corner walls (Figure S1). The chamber surfaces were fully cleaned with ethanol and distilled water and then ozonized (500 ppb ozone) prior to the experiments in order to eliminate the residual reactants on surfaces.

### 2.2 Experimental Design and Procedure

We recruited six young adults, including four females and two males (age: 21–19 years; body mass index 18.4–26.6 kg/m^2^, see Table S1). Prior to each experimental day, the participants were instructed to take an evening shower using supplied soap and shampoo that were free of perfumes and odorants. They were also advised not to use any other personal care products. On the day of the experiment, 30 min before entering the chamber, the participants changed into short-sleeved T-shirts and shorts provided by the researchers. These newly provided garments had been laundered with fragrance-free detergent immediately after purchase and then tumble-dried and sealed individually in zip-lock bags. Personal items were not permitted in the chamber, but the participants could use the tablet computers provided inside the chamber.

We performed experiments under three ACRs with the mixing fans on, namely, 0.5, 1.5, and 3 h^–1^. Additionally, to assess the impact of forced air mixing, we performed an additional experiment at a 3 h^–1^ ACR with the mixing fans off. Each experiment had one replicate, leading to eight experiments in total (Table 1). The detailed procedure of each experiment is shown in Figure S2.

**Table 1:** Ultrafine Particle Deposition Rate, Growth Rate, and Formation Rate in the Experiments^a^

					deposition rate (h^–1^)			growth rate (nm/h)			formation rate (particles/h per person)		
condition	ACR (h^–1^)	inlet ozone (ppb)	indoor ozone (ppb)	ozone loss (ppb)	1–3 nm	3–10 nm	10–20 nm	1–3 nm	3–10 nm	10–20 nm	1–3 nm	3–10 nm	10–20 nm
0.5 h^–1^ ACR, mixing fans on	0.55	226	28	198	0.2	0.1	0.1	0.6	c	c	2.3 × 10^11^	c	c
	0.55	229	30	199	0.4	0.2	0.2	0.4	c	c	1.7 × 10^11^	c	c
1.5 h^–1^ ACR, mixing fans on	1.47	99	24	75	b	0.9	0.8	b	7.1	1.2	b	5.7 × 10^9^	0.8 × 10^9^
	1.49	102	26	76	1.4	1.0	0.8	0.9	6.3	c	4.5 × 10^11^	5.5 × 10^9^	c
3.0 h^–1^ ACR, mixing fans on	2.98	66	25	41	b	2.0	1.8	b	8.7	2.3	b	6.3 × 10^9^	1.2 × 10^9^
	2.98	72	27	45	3.0	2.2	1.7	2.1	7.3	1.7	1.2 × 10^12^	6.5 × 10^9^	1.6 × 10^9^
3.0 h^–1^ ACR, mixing fans off^d^	2.90	67	33	34	1.7	1.0	0.7	2.4	41.3	42.8	7.7 × 10^12^	7.2 × 10^12^	1.4 × 10^12^
	2.94	64	32	32	1.8	0.9	0.5	3.0	41.5	43.2	7.3 × 10^12^	7.2 × 10^12^	1.3 × 10^12^

a The data are reported for three size groups: 1–3, 3–10, and 10–20 nm. Steady-state inlet and indoor ozone levels and ACR are also shown as well as ozone loss (the difference between inlet and indoor ozone). The ACR was calculated by exponential fitting of CO_2_ decay after the participants exited the chamber.

b Data not available due to instrument failure.

c Data not available due to missing particle growth rate caused by negligible particle growth.

d Particle growth rate and formation rate were reported for the first-wave particle formation and growth event.

In essence, the experiments with ACR 3.0 and 1.5 h^–1^ with mixing fans on had the same procedure, consisting of a 3 h morning session without ozone and a 3 h afternoon session with ozone. In both sessions, the participants remained seated at the tables for 90 min after entering the chamber and then stood up to stretch for 10 min, followed by returning to their seats until exiting the chamber. During the lunch break after exiting the chamber, all participants were given the same meal and beverage (a light sandwich with tomato and cheese, along with a bottle of noncarbonated (plain) water). At this time, the chamber was flushed at a high ACR (∼9 h^–1^) to reduce the background level of human-related particles and VOCs.

In scenarios where the mixing fans were switched off, the procedure was similar, except that one participant (no. 6) alternated between the table and the sampling station every 30 min while staying in the chamber. This adjustment aimed to investigate the potential difference in ultrafine particle levels between the bulk air and the peri-human microenvironment (termed the “personal cloud effect”^61−63^).

Experiments with 0.5 h^–1^ ACR lasted for five consecutive hours with ozone present in order to address the slower buildup of reaction products in the chamber air. For all sessions with ozone present, ozone was generated in the supply air duct using a Jelight 600 UV generator (Jelight Co. Inc., USA) and injected 10 min after the participants entered the chamber, targeting a steady-state level of 24–33 ppb inside the occupied chamber (Table 1).

### 2.3 Instrumentation and Quality Control

To measure the particle size distribution in real-time within the initially formed cluster of 1–55 nm and to characterize the ultrafine particle dynamics during experiments, a set of instruments was deployed. 1–3 nm NCA particles were monitored using a Nano Condensation Nucleus Counter (Airmodus A11 nCNC System, Airmodus, Finland) at a sampling flow rate of 2.5 L/min.^49,64,65^ This system consists of a particle size magnifier (PSM A10) and a butanol-based condensation particle counter (CPC A20). The PSM aims to enlarge small particles into a size range that can be detected by the CPC, employing a mixing-type principle. The mixing ratio can be swiftly adjusted (one scan), leading to concurrent size variations in the smallest particles that can be magnified by the PSM. A complete scan included two 2 min periods: the saturator flow climbing from 0.1 to 1.3 L/min (up-scan) and then decreasing back to 0.1 L/min (down-scan). Afterward, we averaged NCA concentrations in the two periods and thus obtained a time resolution of 4 min. Ultrafine particles larger than 3 nm were measured by a set of scanning mobility particle sizer (SMPS) at a sampling flow rate of 0.3 L/min. The SMPS setup included an aerosol charge neutralizer (XRC-05, GRIMM Aerosol Technik, DE), a short differential mobility analyzer (“Vienna” S-DMA, GRIMM), and a condensation particle counter (CPC, model 5416, GRIMM). A full scan lasted for 3 min, resulting in 81 channels in the size range 3–55 nm. Because the instruments were positioned immediately outside the chamber, we sampled the particles with isokinetic core sampling probes at a carrier flow rate of 5 L/min so as to minimize the sampling loss. It is worth nothing that in one experiment conducted at an ACR of 1.5 h^–1^ and another at 3.0 h^–1^ ACR (both with mixing fans on), the A11 nCNC experienced a failure, and thus, we did not successfully collect NCA data from these two experiments.

The ozone concentration inside the chamber was monitored with an ozone monitor (model 724, Tanabyte, US) at a 1 min interval and 2.0 L/min sampling flow rate. Another ozone monitor of the same model measured the inlet ozone level at the supply diffuser (Figure S1). The difference between inlet and indoor ozone level represents the ozone loss inside the chamber (ppb), which is an indicator of gaseous ozone byproduct abundance when ozone removal indoors is dominated by ozone loss on indoor surfaces (including humans).^66^ We also measured real-time CO_2_ level (HOBO MX1102, Onset Inc., US) at two locations inside the chamber (Figure S1).

In addition, we monitored mixing ratios of indoor VOCs using a Vocus proton transfer reaction time-of-flight mass spectrometer (Vocus PTR-ToF-MS, Tofwerk AG and Aerodyne Research, Inc.) to capture the gas-phase products from ozone–human chemistry. During the experiment period, the ionization source pressure was regulated to 2.0 mbar. Every 4 s, a mass spectrum was collected in the range 11–500 Th. The mass resolution was ∼10,000 at m/Q 500. The instrument sampled using 0.65 m 14′ perfluoroalkoxy (PFA) tubing at the flow rate of ∼100 sccm from the main stream air line (1/2″ PFA), which was driven at 12.5 L/min by an external pump either from the chamber or from the supply air. We calibrated the instrument before, during, and after the campaign (in total 4 times) using a gas mixture standard (details in Section S1).

Prior to the experimental campaign, all instruments were fully serviced and calibrated. As seen in Table 1, each experiment had one replicate, with differences generally within 15%, indicating good reproducibility of the experiment results.

### 2.4 Data Analysis

The real-time NCA concentrations were obtained by inversing the raw data using the stepwise method,^67^ and then grouping into four size bins (1.28–1.74, 1.74–1.99, 1.99–2.15, and 2.15–3.33 nm). Considering the relatively higher background level of >20 nm ultrafine particles in the chamber, we focused on particle data within the size range of 3–20 nm from the SMPS for quantitative analysis, including 54 size bins. Afterward, we calculated the per-person formation rate of ultrafine particles based on the material balance inside the chamber
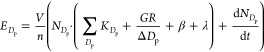
1where *E*_*D*_p__ is the per-person ultrafine particle formation rate (particles/h per person), including all source terms, such as nucleation and growth from smaller size particles; *V* is the chamber volume (m^3^); *n* is the number of occupants in the chamber (-); *N*_*D*p_ is the particle concentration for a specific size *D*_p_ (#/cm^3^); ∑_*D*p_*K*_*D*_p__*N*_*D*_p__ is the net coagulation sink rate (h^–1^) for the particle population including size *D*_p_;^19,68^ GR is the growth rate (nm/h) within the size range of Δ*D*_p_, the calculation procedure of which is explained in Section S2; β is the deposition rate obtained via exponential fitting of the particle number concentration during the decay period in each experiment after the participants exited the chamber (h^–1^); λ is the ACR (h^–1^) obtained by exponential fitting of CO_2_ decay after the participants exited the chamber; and  is the particle concentration change rate. For a robust estimation, we grouped the particle data into three size bins: 1–3, 3–10, and 10–20 nm to report their GR and consequently formation rates. Hence, Δ*D*_p_ corresponded to 2, 7, and 10 nm, respectively. For experiments with mixing fans on, *E*_*D*_p__ was calculated at steady state, whereas in experiments with mixing fans off, the *E*_*D*_p__ was obtained by curve fitting using the first particle formation and growth event, for which the GR was calculated (Section S2).

We also analyzed the correlations between ultrafine particle concentrations (1–3 and 3–20 nm) and VOC levels (707 detected signals) with the Pearson method to explore the relationship between particles and gas-phase products from ozone–human chemistry. Two types of correlation data were used: (1) quasi-steady-state concentrations (the average of the last 30 min of the occupied period) of particles and VOCs during ozone–human reaction across all the experiments with mixing fans on and (2) time-series data of particles and VOCs in mixing-fan-off experiments. Special attention was given to marker VOCs for ozone–human chemistry, such as 6-MHO, 4-OPA, and OH-6MHO,^39^ and their levels were strongly correlated with particle concentrations (Pearson |*r*| > 0.8 and *p* < 0.05).

## 3 Results and Discussion

### 3.1 Time Series of Ultrafines in Relation to Ventilation

**Figure 1:**
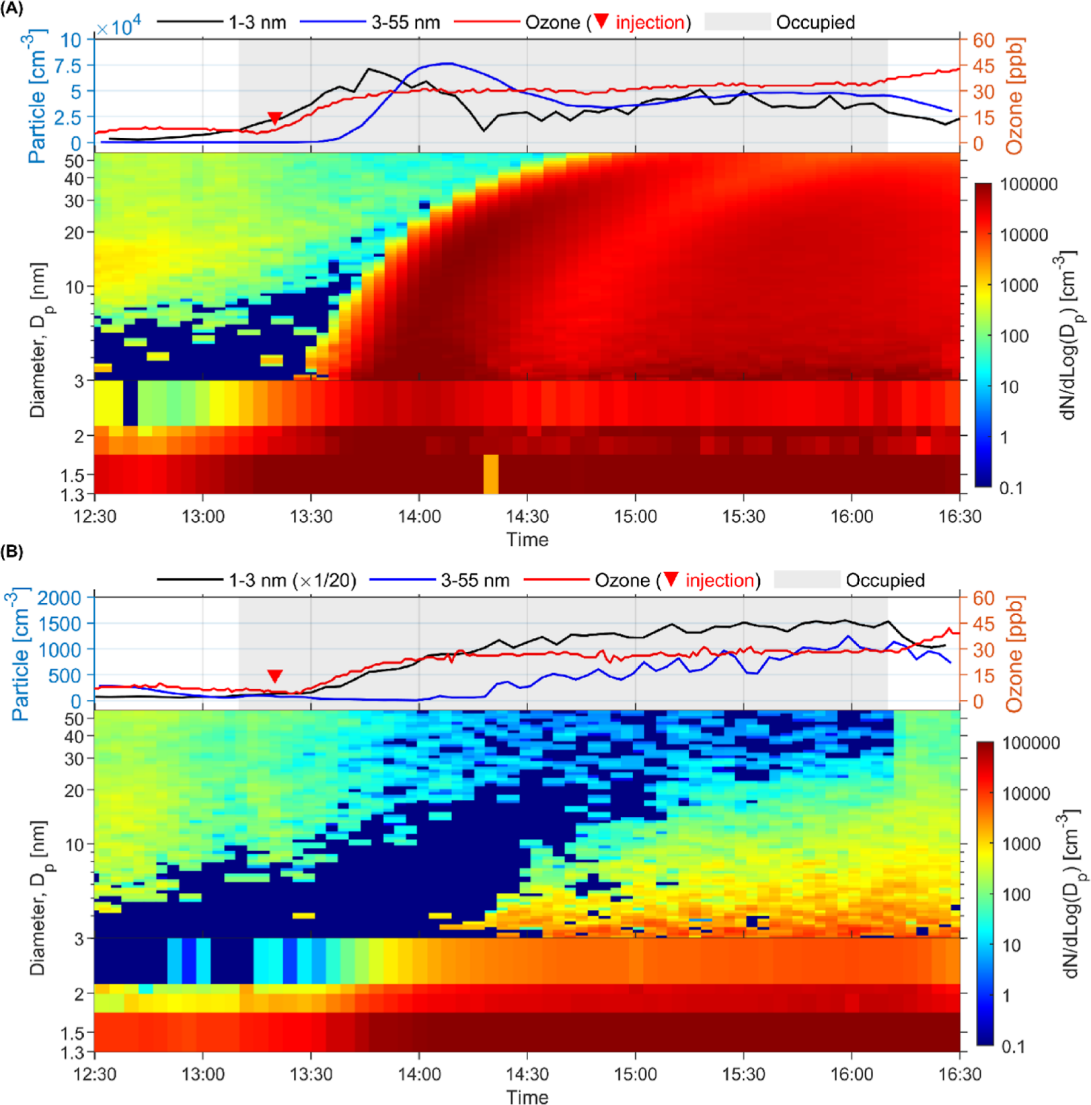
Time series of ultrafine particle formation from ozone–human chemistry and subsequent growth at 3.0 h^–1^ ACR with (A) mixing fans off and (B) mixing fans on. 1–3 nm particles were measured by the A11 nCNC system, and the diameter was activation size, whereas 3–55 nm particles were measured by SMPS, and the diameter was mobility size. Particle level (left axis) is presented in number concentration (particles per cm^3^, i.e., cm^–3^). Shaded area in the top charts indicates the time when the chamber was occupied; and the upside-down triangle represents the moment when ozone was injected into the chamber. Note that in the top chart of (B), the 1–3 nm particle concentrations were divided by 20.

Figure 1A shows a time series of the ozone mixing ratio and ultrafine particle size distributions in the occupied chamber in the afternoon session in the mixing-fan-off experiment with an ACR of 3.0 h^–1^. When participants entered the chamber, there was a slight increase of NCA levels, presumably because of the background ozone (∼6 ppb). When ozone injection started, the ozone level inside the chamber gradually climbed and then reached a steady state of 33 ppb. In parallel, the NCA concentration sharply increased―starting with the smallest size (1.28–1.74 nm) and followed sequentially by the larger sizes. We observed a 20 min delay for the growth of >3 nm ultrafine particles relative to NCAs. A typical “banana-like” particle size evolution was witnessed, demonstrating the particle growth process during the formation via ozone–human chemistry. Owing to the avoidance of personal care products and control of participants’ clothing, terpene levels (such as limonene and alpha-terpene) remained low (<0.1 ppb) and thus negligibly contributed to particle formation in the presence of ozone. Although human-exhaled air also contains VOCs that can react with ozone, our previous study has demonstrated that these reactions do not contribute to particle generation, and the predominant mechanism is ozone reaction with human skin lipids.^49^ Ozone can oxidize unsaturated compounds in human skin lipids (such as squalene^39^), which generates highly oxygenated semivolatile organic compounds (SVOCs) through peroxy radical reactions including autoxidation.^69^ Molecular clusters of these oxygenated species then nucleate to form NCA and to initiate the following particle growth, which can be stabilized and enhanced by human-emitted NH_3_.^49,70^

Interestingly, around 1 h after the first wave of particle generation, we noticed another particle formation and growth event (from 14:30 in Figure 1A) but with substantially lower ultrafine particle levels relative to those of the first one. Rai et al.^46^ also observed such a multiwave generation of ultrafine particles (>10 nm) in the experiments of ozone reaction with skin-oiled clothing, although not in the case of high ACR (2.7 h^–1^) owing to less available reactants being present relative to those in this study (one skin-oiled T-shirt vs six human participants). This phenomenon can be caused by particle dynamics related to generation, growth, condensation, and deposition.^71^ The primary burst was mainly attributed to the nucleation of hypothetical SVOC vapors generated from ozone reaction with human skin lipids (such as squalene).^49^ These freshly formed clusters then acted as sites for SVOC vapor condensation, causing a decrease in the SVOC vapor concentration below the nucleation threshold and resisting further nucleation events. Subsequently, condensation became the dominant process, leading to particle size growth and the uptake of SVOC vapor. Moreover, the particle count inside the chamber also decreased as nucleation ceased, and particles were continuously removed through ventilation and deposition (Figure 1A). Consequently, the pool of available condensation sites for SVOC vapor decreased, leading to accumulation of SVOC vapors to form new clusters and subsequently causing the ultrafine particle concentration to rise once more, resulting in another wave of particle growth. Another potential explanation for the second formation event is the nucleation and partitioning of some secondary gaseous products from ozone–human chemistry to a solid phase, such as carboxylic acids (see Section 3.3),^72−74^ some of which can consequently decompose into acetic and formic acids.^73,75^ Such ultrafine particle formations were not observed in the morning session without ozone (Figures S3 and S4).

The ultrafine particle formation changed when the mixing fans were turned on (Figures 1B and S5–S6). We witnessed the formation of NCA inside the chamber after ozone injection, but the concentration gradually reached a steady state instead of waving. The steady-state particle concentration was much lower than that for the mixing-fan-off scenario (3.0 × 10^4^ vs on average 5.0 × 10^4^ particles/cm^3^). The reduction in particle generation was more obvious for >3 nm particles: a growth showed up 1 h after ozone injection and then slowly reached steady state at a considerably lower level (0.1 × 10^4^ vs on average 5.9 × 10^4^ particles/cm^3^). We did not observe the typical “banana-like” particle growth. Although increased air velocity may increase the chance of particle collision that potentially contributes to particle growth, the activation of the mixing fans strongly increased the deposition of ultrafine particles (Table 1) and also likely the SVOC vapors^76^ inside the chamber, thus limiting the nucleation and condensation processes. The increased deposition could be caused by two factors. (1) At the chamber scale, the operation of mixing fans elevated the average air velocity inside the chamber, which may have resulted in increased particle and SVOC deposition rates.^77−79^ Upon assessing the influence of chamber surface materials on particle deposition, it was found to be negligible due to the overriding impact of air velocity compared to that of surface properties.^80^ (2) At the localized areas where the fans operated, they drove a considerable amount of SVOC vapors and newly formed particles toward the corner walls, essentially acting as air purifiers. In addition, the ozone loss also increased when the mixing fans were on, from 33 to 43 ppb on average (Table 1), leading to a 6 ppb reduction of steady-state ozone inside the chamber. The reduced ozone level may also be attributed to the fact that intensified air movement transported more ozone to surfaces where it could readily react. It is worth mentioning that the NCA level in this experiment was 2 orders of magnitude higher than that in our previous study (60–300 particles/cm^3^),^49^ although the ACRs were similar (both around 3 h^–1^). Such a disparity may be due to the difference in the chamber setup (fully stainless-steel vs vinyl floor), environmental conditions (relative humidity 20 vs 50%; steady-state ozone level 40 vs 26 ppb), indoor level of organic and inorganic chemicals (such as NO_*x*_ and SO_*x*_, which play important roles in particle formation^81^), and the sensitivity of the instrument.^82^ This disparity highlights the complexity of the gas-to-particle conversion processes. Nevertheless, in both studies, NCA formation occurred only when ozone was injected into the occupied chamber, and the time-series of NCA followed that of ozone, which illustrated NCA formation via ozone–human chemistry.

**Figure 2:**
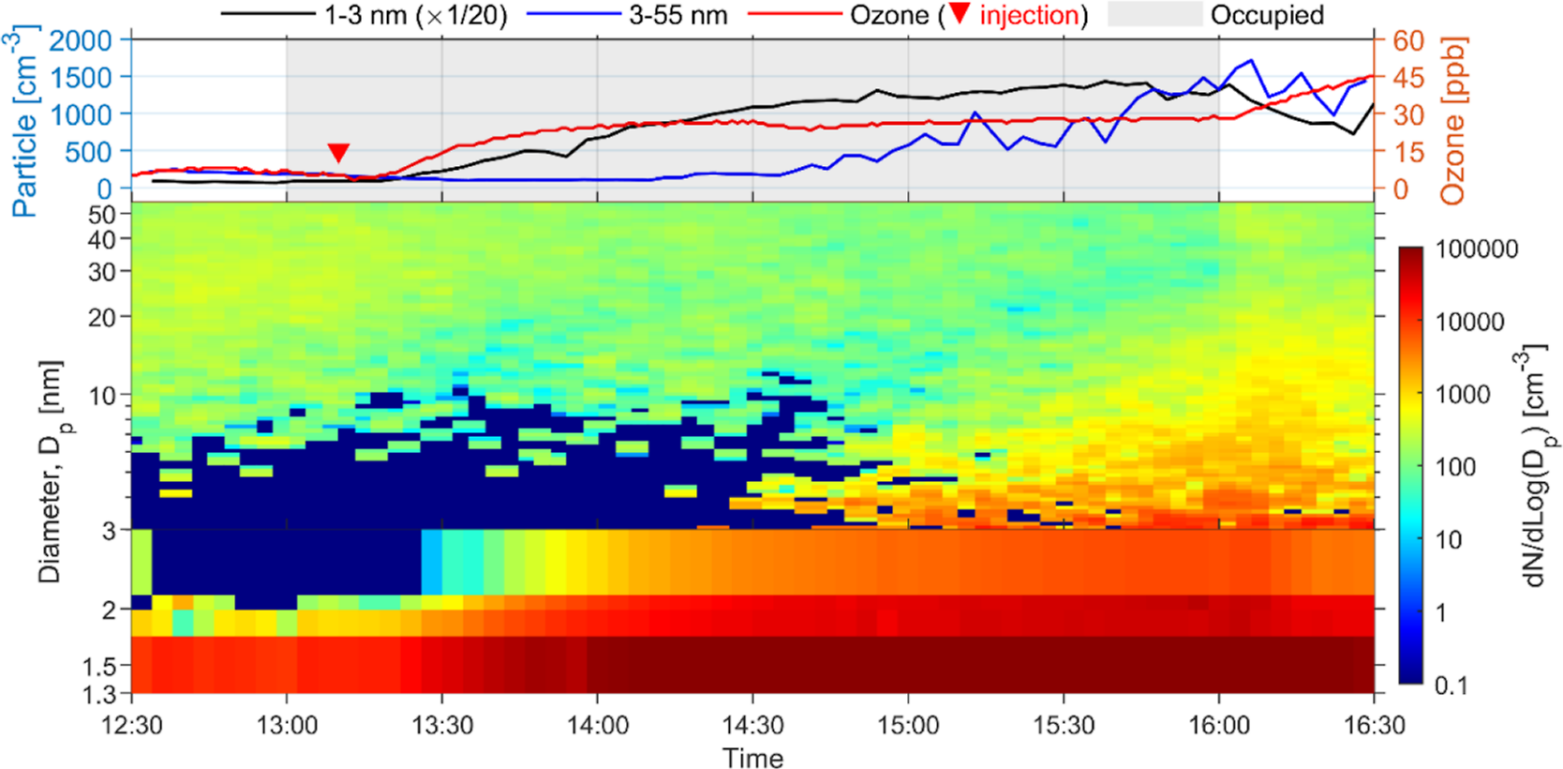
Ultrafine particle formation from ozone–human chemistry at 1.5 h^–1^ ACR with mixing fans on. 1–3 nm particles were measured by A11 nCNC system, and the diameter was the activation size, whereas >3 nm particles were measured by SMPS, and the diameter was the mobility size. Particle level (left axis) is presented in number concentration (particles per cm^3^, i.e., cm^–3^). Shaded area in the top chart indicates the time when the chamber was occupied; and the upside-down triangle represents the moment when ozone was injected into the chamber. Note that in the top chart, the 1–3 nm particle concentration was divided by 20.

When the ACR was reduced to 1.5 h^–1^ with the mixing fans on, the particle formation behavior shared similar trends as that at 3.0 h^–1^ (Figures 2, S7 and S8). The time lag between the formation of NCA and >3 nm particles was longer (∼1.5 h), indicating a slower particle growth (Table 1). Although the steady-state levels of ultrafine particles at 1.5 h^–1^ were similar to that at 3.0 h^–1^, we observed lower generation rates at lower ACR values (Table 1).

**Figure 3:**
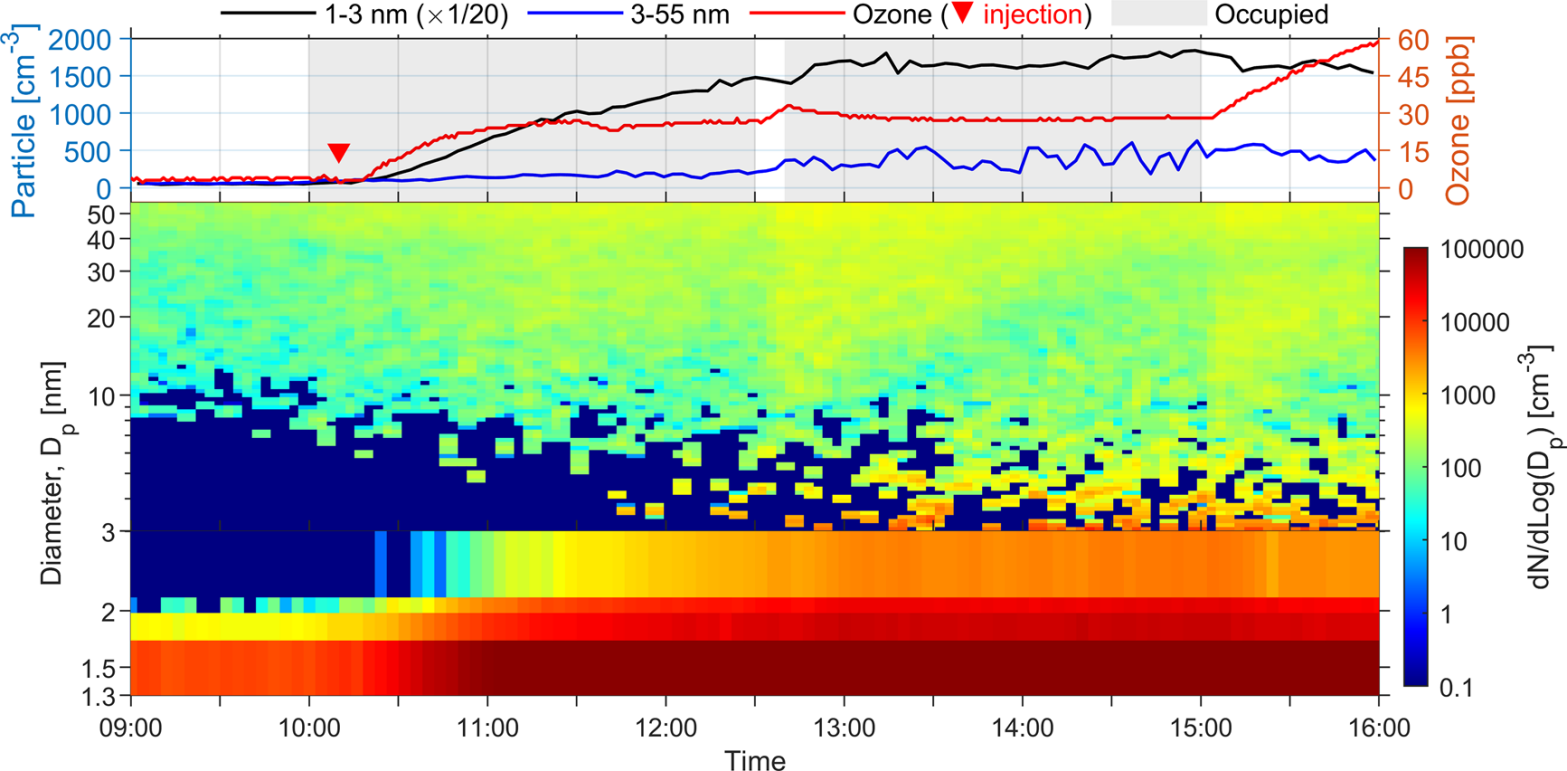
Ultrafine particle formation from ozone–human chemistry at 0.5 h^–1^ ACR with mixing fans on. 1–3 nm particles were measured by A11 nCNC system, and the diameter was activation size, whereas >3 nm particles were measured by SMPS, and the diameter was mobility size. Particle level (left axis) is presented in number concentration (particles per cm^3^, i.e., cm^–3^). The shaded area in the top chart indicates the time when the chamber was occupied; and the upside-down triangle represents the moment when ozone was injected into the chamber. There was a 10 min bathroom break at 12:30. Note that in the top chart, the 1–3 nm particle concentration was divided by 20.

We did not observe meaningful particle growth beyond 3 nm when the ACR was further reduced to 0.5 h^–1^ with the mixing fans were on (Figures 3 and S9). This is different from the observation of Rai et al.,^46^ where a considerable generation of particles from ozone reaction with skin-oiled clothing was witnessed at 0.5 h^–1^ but without forced air mixing (fans). The operation of the mixing fans suppressed particle growth, as discussed previously. A potential contributor to the limited particle growth at the lowest ACR was the relatively higher concentration of larger particles (>20 nm) when ozone was present inside the chamber (on average 107 cm^–3^ at 0.5 h^–1^ ACR vs 53 and 5 cm^–3^ at 1.5 and 3.0 h^–1^ ACR, respectively). Although the coagulation sink effect of these background particles on newly formed clusters (1 × 10^–2^ h^–1^ at 0.5 h^–1^ ACR vs 4 × 10^–4^ h^–1^ at 3.0 h^–1^ ACR) was negligible relative to ACR and deposition rate, these particles can act as a sink for SVOC vapors, which may hinder particle formation and further growth.

### 3.2 Ultrafine Particle Dynamics and Formation Rates

Table 1 lists ultrafine particle formation rates from ozone–human chemistry and their dependence on the ACR and fan operation. It can be clearly seen that particle deposition rates of all size bins increased with the increase of ACR.^83,84^ The lower particle deposition rates when the mixing fans were off demonstrated the profound effect of fan operation. The NCA (1–3 nm) deposition rates obtained in this study were generally at the lower bound of the previously reported values.^49,77^ This observation may indicate that after the participants exited the chamber, there could be ongoing NCA generation inside, potentially owing to ozone gas-phase chemistry or reaction with surfaces contaminated with skin lipids.^85^ Ozone loss decreased with increasing ACR.^66^

The NCA growth rates also increased with ACR. The values obtained in this study agreed well with those reported for outdoor conditions.^86−88^ For >3 nm particles, the growth rates in the mixing-fan-off scenario (41.3–43.2 nm/h) were comparable to those associated with particle formation from ozone reaction with painting materials (33.9 ± 9.1 nm/h)^25^ and much lower than that from ozone–limonene reaction (6300 nm/h).^89^ Per person particle formation rates increased with ACR as well. The NCA formation rate per person was 40–95× higher than that in our previous study (ranging 1.3–3.0 × 10^5^ million particles/h per person),^49^ also with participants wearing short clothing and ∼3 h^–1^ ACR with mixing fans on. Potential reasons for this disparity are discussed in Section 3.1. When the mixing fans were off, the NCA formation rate further increased by 6×, though it remained an order of magnitude lower than emissions from cooking^90^ and the use of 3D printers.^68^ The remarkable increase of >3 nm particle formation rates by 3 orders of magnitude relative to that in the mixing-fan-on scenario further demonstrates the importance of indoor air flow dynamics for ultrafine particle formation and growth.

### 3.3 Correlations between Ultrafine Particles and VOCs

**Figure 4:**
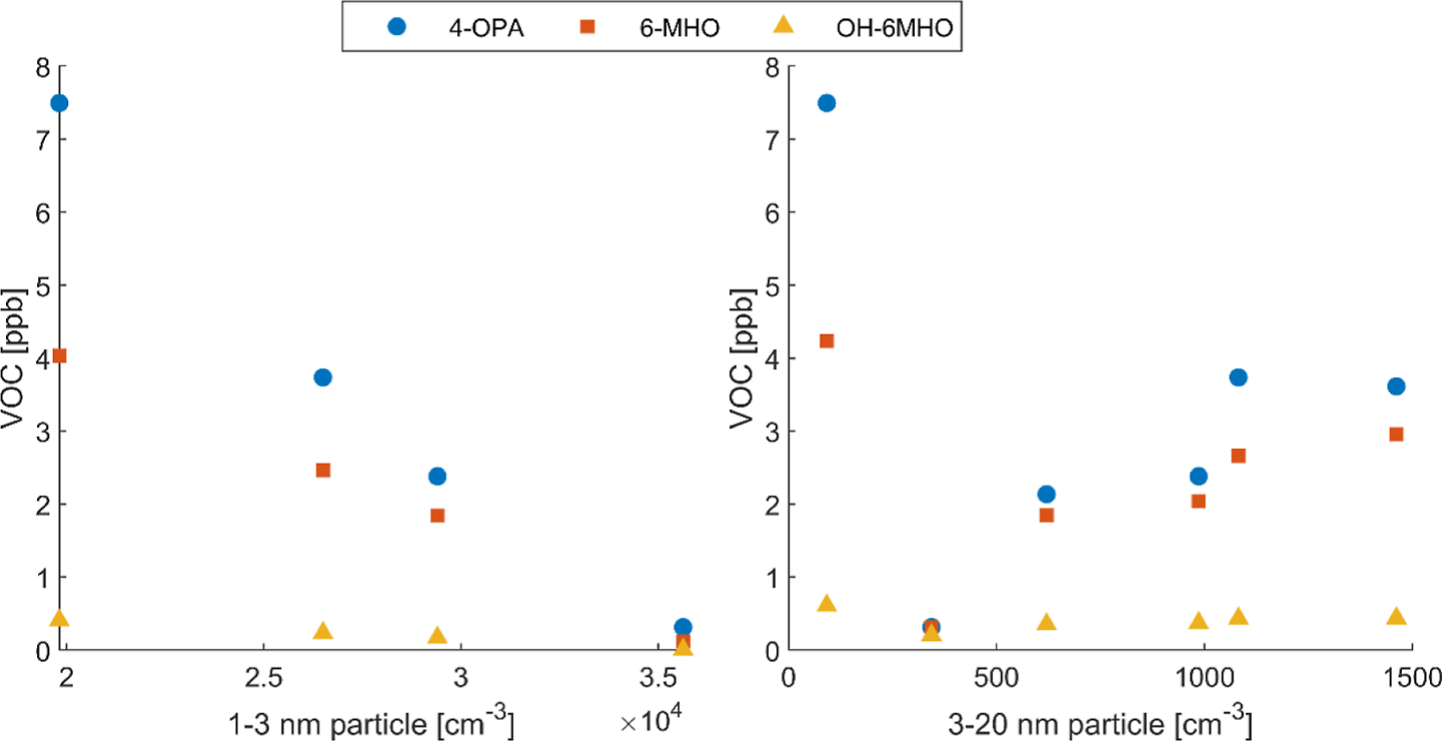
Correlation between quasi-steady-state concentrations of particles and VOCs (4-OPA, 6-MHO, and OH-6MHO) during ozone–human reaction across all the experiments with mixing fans on. 1–3 nm particles had four data series available due to an instrument failure, whereas 3–20 nm particles have full data sets of six experiments. Particle level (bottom axis) is presented in number concentration (particles per cm^3^, i.e., cm^–3^).

We first examined the correlation between quasi-steady-state concentrations of particles and VOCs during ozone–human reaction across all the experiments with mixing fans on. As expected, owing to the relatively small sample size (*N* = 4 for 1–3 nm and *N* = 6 for >3 nm), we did not find VOCs with signals that were strongly correlated with ultrafine particle concentrations. However, when looking at the correlations between particles and specific VOCs from ozone–human chemistry, namely, 4-OPA, 6-MHO, and OH-6MHO, we found negative correlations with 1–3 nm NCA particles, which are opposite to the results of our previous study.^49^ The disparity may simply be due to the much smaller sample size in this work (4 vs 15 experiments). Another potential explanation is that in the previous study, the ACR was fixed at 3.2 h^–1^, and thus, the variations in NCA generation were mainly attributed to the amount of reactants (various skin surface areas) and environmental conditions (air temperature, humidity, and onset of ozone dosing), both associated with reaction strength and VOC levels. Therefore, a positive correlation was observed between the nucleation and VOC gases. This study, on the contrary, applied the same experimental conditions (apart from ventilation) and was thus assumed to have relatively constant reaction strength between ozone and human skin lipids.^49^ The main difference lies in the transport limitations of ozone and the low-volatility products attributed to ventilation.^54^ The only variable, ACR, may play a role in driving the competition between nucleation and gas emissions, as has been discussed in Section 3.1 (Figure 1A), and thus lead to a negative correlation. On the other hand, 3–20 nm particles showed generally positive correlations with the VOC markers (Figure 4), probably indicating the contribution of the reactions generating these VOCs to the growth of particles. In addition, the quasi-steady-state particle concentrations were not correlated with ozone loss. Ozone loss may not be a good surrogate for indoor nanoparticle levels generated from ozone–human chemistry (Figure S10). This can be owing to the sensitivity of the nanoparticle yield to the pre-existing level of indoor particles and their chemical properties and to the particle deposition altered by surface properties and airflow field.^66^ Nevertheless, ozone loss times ACR, equivalent to the chamber ozone concentration × the total rate constant for ozone removal on human and chamber surfaces (*k*_sum_), were positively correlated with particle formation rates (Figure S10). At a constant surface-to-volume ratio (A/V), ozone flux to surfaces scales with the product of the chamber ozone concentration and *k*_sum_. This observation may thus indicate that a larger ozone flux to occupant surfaces results in higher particle generation. Nevertheless, given the limited sample size, the results should be cautiously treated.

**Figure 5:**
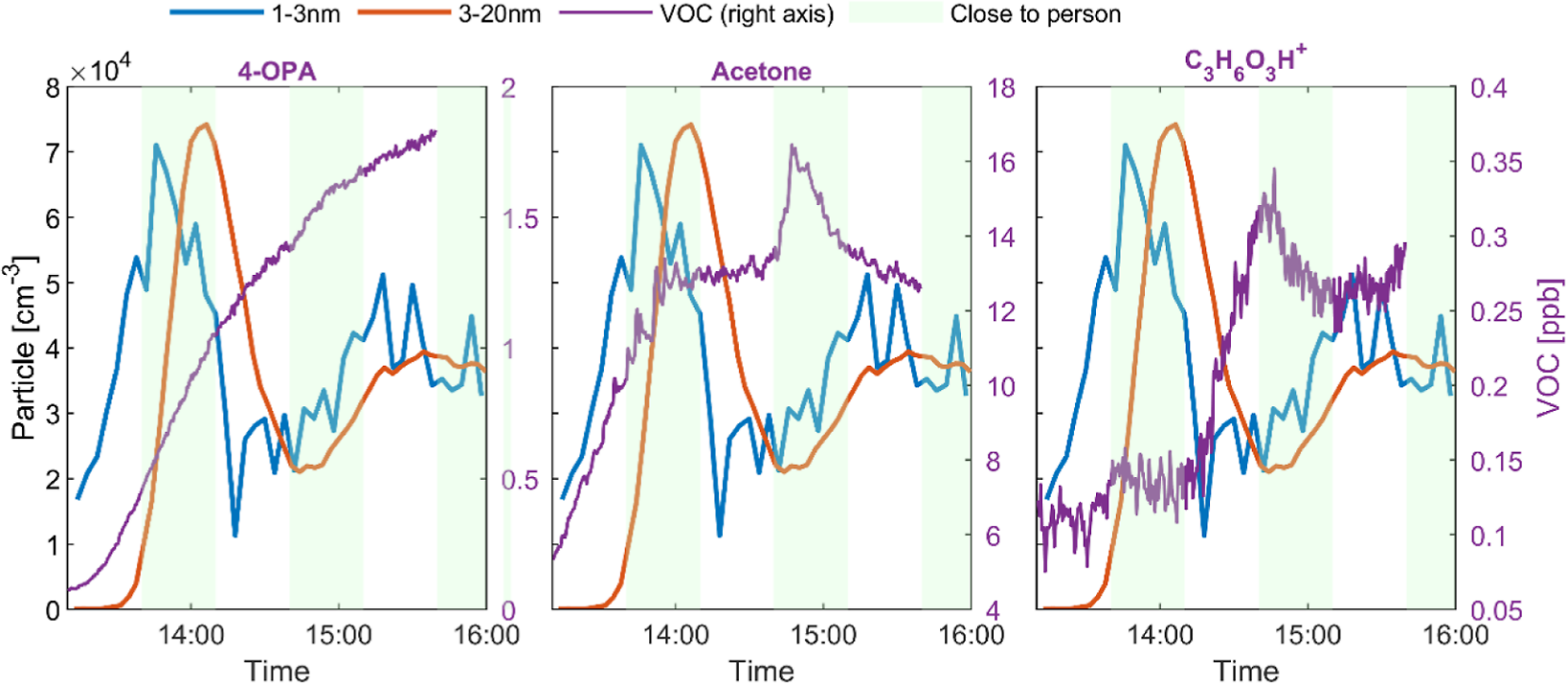
Time-series profiles of 1–3 and 3–20 nm ultrafine particles, and three representative VOCs: 4-OPA (left), acetone (middle), and C_3_H_6_O_3_(right). Pearson |*r*| > 0.8 for acetone and C_3_H_6_O_3_ with statistical significance. The shaded area represents the duration when one participant (no. 6) moved to the sampling station, and thus, the particle and VOC measurements were in the peri-human microenvironment. Particle level (left axis) is presented in number concentration (particles per cm^3^, i.e., cm^–3^).

Figure 5 shows the time-series profiles of the ultrafine particles and three representative VOCs: 4-OPA, acetone, and C_3_H_6_O_3_. While ultrafine particles underwent the two-wave formation (see also Figure 1A), 4-OPA, mainly generated by secondary reactions,^39^ kept climbing during the whole experiment, and the profile was not disturbed by the sampling location. This echoes the finding from literature that secondary products tend to distribute in the room instead of being confined in the peri-human (near-body) microenvironment.^91^ Acetone, originating from both ozone–human chemistry and human exhalation,^41,92^ showed some waving characteristics, similar to those of particles. However, such a wave was mostly related to the sampling location, as evidenced by the sudden peak when the participant moved to the sampling station. When the participant sat at the sampling station for the first time, acetone inside the chamber was in the climbing stage and had not yet reached a steady-state. Hence, the potential increase due to the participant’s moving could be assimilated into the overall rising trend. During the second instance, the acetone concentration had almost reached a steady state, so the influence of the nearby participant was thus more pronounced. C_3_H_6_O_3_, another compound that was strongly correlated with particle concentration variations, started to increase after the first wave of nucleation and then declined during the second particle formation event. This may be a sign that this compound was involved in particle formation and growth processes. Although we were not able to know the exact structure of the compounds, we suspect C_3_H_6_O_3_ may be a carboxylic acid, a similar highly oxidized chemical, or fragment thereof, which has the potential to contribute to the secondary burst of particles,^72−74^ as has been discussed in Section 3.1 (Figure 1A). These correlation analyses reflect the complicated relationship between the particles and gas-phase products initiated from ozone–human chemistry. Additionally, regarding the personal cloud effect, it is difficult to draw conclusions about the difference in particle levels between the bulk air and the peri-human microenvironment. This is the case because of the fluctuation of NCA levels in general and because changing the proximity of the sampling location in relation to the person coincided with various particle formation and growth events.

### 3.4 Limitations

Several limitations should be acknowledged when interpreting these results. This study used the stepwise method for data inversion of NCA. Chan et al. proposed that this method might overestimate NCA concentrations in scenarios with high levels of larger particles and strong concentration fluctuations.^93^ While the experiments with the mixing fans on generally had stable and relatively low particle concentrations, this overestimation issue might apply to the mixing-fan-off experiments in this study. Due to limited resources, we performed only one replicate for each experiment. Nevertheless, the observed differences were generally within 15%, indicating a high level of reproducibility. In addition, we only performed mixing-fan-off experiments at 3.0 h^–1^ ACR. We suspected that without mixing fans particle formation may be more pronounced at lower ACR. As discussed above, the deposition rates of ultrafine particles, obtained by exponential fitting of the particle decay data in the unoccupied chamber, were generally low. Given the generally low particle concentrations, the calculated contribution of particle coagulation to particle decay was less than 10%. However, it remains uncertain whether the residual reactions inside the chamber formed ultrafine particles. Therefore, it is doubtful whether the ultrafine particle decay rates can represent deposition loss rates and may thus bring further uncertainties to the calculated formation rates. Finally, we are currently unable to identify the compound C_3_H_6_O_3_, which could be potentially linked to particle growth. Comprehensive findings on various VOCs, including the influence of ventilation and air mixing, will be presented in a future paper.

Another limitation of this study is the lack of measurements encompassing the full size range of ultrafine particles (1–100 nm), especially in the mixing-fan-off experiments, where we expected particle growth beyond 50 nm. In experiments investigating the influence of ACR, the mixing fans were on, making it challenging to observe meaningful particle growth, to which future research should pay attention to. In addition, the steady-state ozone levels in this study varied narrowly within 24–33 ppb, in accordance with that measured in buildings during ozone pollution episodes.^94^ Given the varied indoor ozone levels in buildings,^95−97^ future research of ozone–human chemistry at various ozone concentrations is warranted. In addition, this study lacked a detailed measurement of the air velocity inside the chamber. Future research can consider measuring air velocities at multipoints or simulating airflow field using computational fluid dynamics to investigate the relationship between air movement and indoor nanoparticle dynamics. Finally, future studies should consider more robust approaches to examine the ultrafine particle and ozone distribution around humans generated from ozone–human chemistry, such as multipoint measurements at various points in a room and in the peri-human microenvironment.^62^

### 3.5 Implications

The results of this study emphasize that the operation of mixing fans can effectively restrain particle formation and growth. Consequently, research conducted with the use of mixing fans may substantially underestimate the occurrence of particle formation and consequently misinterpret particle levels and source strengths when applying the results to real indoor environments. It is noteworthy that the mixing-fan-off experiments in our study had acceptable air mixing: the disparity in CO_2_ levels recorded at two distinct locations was within 15%, whereas the one with mixing fans on was within 5%. In light of these observations, a better design and operation of the ventilation supply exhaust system can be considered a priority relative to using mixing fans in experiments dealing with airborne particle dynamics. The difference in the behaviors of CO_2_ and particles under mixing-fan-on and -off scenarios also implies that CO_2_ may not be a reliable indicator of indoor air quality, especially with regard to particles originating from indoor chemistry.

As discussed above, the operation of mixing fans suppressed particle growth, likely by enhancing the deposition of newly formed particles and SVOC vapors via elevated air velocity and by impacting them on walls (similar to an effect of air purifiers). The results could be altered if the orientations of the fans were different. Nevertheless, the findings may imply that ventilation and indoor air movement may have a more significant influence on particle dynamics and fate relative to indoor chemistry. Given the common use of fans in buildings, the impact of fan operation on indoor chemistry and ultrafine particle dynamics in real buildings merits closer attention.

The influence of ventilation on indoor ultrafine particle levels generated from ozone–human chemistry in real buildings can be more intricate than that in the scenarios investigated in this study. For instance, this study maintained similar ozone levels across all of the experiments. However, in real buildings, ozone levels change with ventilation, with consequences for indoor chemistry and for the concentrations of ozone reaction products. Elevated ventilation rate can introduce more ozone indoors and thus increase the concentrations of ozone that react with humans and other indoor surfaces. In turn, it can lead to higher dilution of the resulting reaction products and to lower ozone loss at a given outdoor ozone level, which can be considered an indicator for ozone byproducts.^66^ On the other hand, increased ventilation also introduces more ultrafine particles into the building, potentially limiting the nucleation of new particles in indoor air. Therefore, more laboratory and field experiments are encouraged to explore the on-site influence of ventilation rate and ventilation techniques on ozone–human chemistry and the associated ultrafine particle behavior in buildings.

Ozone–human chemistry generates both gaseous and particulate products. As indicated in this study, the two can be (a) “competitive”: where there is a shift from gaseous to particulate reaction products through nucleation and consumption of gas molecules; (b) “collaborative”: with positive correlations between particle growth and several marker VOCs; and (c) “independent”: featuring distinct dynamic profiles of particle and VOC variations. A better understanding of these processes requires further investigations through simultaneous measurements of ultrafine particles and VOCs in ozone–human chemistry experiments, combined with theoretical and mechanistic analyses of the physicochemical processes at play. Such investigations can include a modeling framework to further investigate particle dynamics generated from ozone–human chemistry, especially in various indoor conditions. These investigations stand to benefit from the insights gleaned from this study. Additionally, the chemical composition and the consequent health effects of ultrafine particles generated by ozone–human chemistry require additional research.

## Supporting Information Available

Additional experimental details related to measurement of VOCs, methodology for estimating particle growth rate, schematic layout of the climate chamber with sampling locations, illustration of experiment procedures, time series of ultrafine particle concentrations and size distributions at 3.0 h^–1^ ACR with mixing fans off, time series of ultrafine particle concentrations and size distributions at 3.0 h^–1^ ACR with mixing fans off (replicate), time series of ultrafine particle concentrations and size distributions at 3.0 h^–1^ ACR with mixing fans on, time series of ultrafine particle concentrations and size distributions at 3.0 h^–1^ ACR with mixing fans on (replicate), time series of ultrafine particle concentrations and size distributions at 1.5 h^–1^ ACR with mixing fans on, time series of ultrafine particle concentrations and size distributions at 1.5 h^–1^ ACR with mixing fans on (replicate), time series of ultrafine particle concentrations and size distributions at 0.5 h^–1^ ACR with mixing fans on (replicate), correlation between quasi-steady-state concentrations of particles and ozone loss and between particle emission rates and ozone removal rates, and physiological data of participants in the experiments (PDF)
