# Supplementary material for: Influence of Ventilation on Formation and Growth of 1–20 nm Particles via Ozone–Human Chemistry
Source: Environ Sci Technol. 2024 Feb 7;58(10):4704–15. doi: 10.1021/acs.est.3c08466 (PMC10938884; doi:10.1021/acs.est.3c08466)
Supplement: Supplementary file 1 — es3c08466_si_001.pdf [file es3c08466_si_001.pdf]

Supporting Information for

Influence of ventilation on formation and growth of 1-20 nm particles via ozone-human chemistry

Shen Yang,[1] Tatjana Mueller,[2] Nijing Wang,[2] Gabriel Bekö,[3] Meixia Zhang,[1,4] Marouane Merizak,[1] Pawel Wargocki,[3] Jonathan Williams,[2,5] Dusan Licina[1]*

Affiliations
[1]Human-Oriented Built Environment Lab, School of Architecture, Civil and Environmental Engineering, École Polytechnique Fédérale de Lausanne (EPFL), 1015 Lausanne, Switzerland
[2]Max Planck Institute for Chemistry, Hahn-Meitner Weg 1, 55128 Mainz, Germany
[3]International Centre for Indoor Environment and Energy, Department of Environmental and Resource Engineering, Technical University of Denmark, 2800 Kongens Lyngby, Denmark
[4]School of Mechanical Engineering, Beijing Institute of Technology, Beijing 100081, China
[5]Energy, Environment and Water Research Center, The Cyprus Institute, 2121 Nicosia, Cyprus

*Corresponding authors. Email: dusan.licina@epfl.ch

Total number of pages: 14
Total number of figures: 12
Total number of tables: 1

This Supporting Information file includes:

Section S1. Additional experimental details related to measurement of VOCs
Section S2. Methodology for estimating particle growth rate
Figure S1. Schematic layout of the climate chamber with sampling locations
Figure S2. Illustration of experiment procedures
Figure S3. Time series of ultrafine particle concentrations and size distributions at 3.0 h^{-1} air change rate with mixing fans off
Figure S4. Time series of ultrafine particle concentrations and size distributions at 3.0 h^{-1} air change rate with mixing fans off (replicate)
Figure S5. Time series of ultrafine particle concentrations and size distributions at 3.0 h^{-1} air change rate with mixing fans on
Figure S6. Time series of ultrafine particle concentrations and size distributions at 3.0 h^{-1} air change rate with mixing fans on (replicate)
Figure S7. Time series of ultrafine particle concentrations and size distributions at 1.5 h^{-1} air change rate with mixing fans on
Figure S8. Time series of ultrafine particle concentrations and size distributions at 1.5 h^{-1} air change rate with mixing fans on (replicate)
Figure S9. Time series of ultrafine particle concentrations and size distributions at 0.5 h^{-1} air change rate with mixing fans on (replicate)
Figure S10. Correlation between quasi-steady-state concentrations of particles and ozone loss and between particle emission rates and ozone removal rates
Table S1. Physiological data of participants in the experiments

Section S1. Additional experimental details related to measurement of VOCs

We monitored mixing ratios of indoor VOCs using a Vocus proton transfer reaction time-of-flight mass spectrometer (Vocus PTR-ToF-MS, Tofwerk AG and Aerodyne Research, Inc.) to capture gas-phase products from ozone-human chemistry. Any VOC having proton affinity higher than water can undergo proton transfer reactions with water and be detected at its protonated mass by a mass spectrometer.[1] Compared to previous PTR instrumentation, Vocus PTR uses a new reagent ion source and a focusing ion–molecule reactor (FIMR), which significantly improves the sensitivity and detection efficiency.[2] During the experiment period, the ionization source pressure of the Vocus PTR was regulated to 2.0 mbar. Every 4s, a mass spectrum was collected in the range of 11-500 Th. The mass resolution was ~ 10000 at m/Q 500. The Vocus PTR sampled side stream air using 0.65 m ¼' perfluoroalkoxy (PFA) tubing at the flow rate of ~ 100 sccm from the main inlet (1/2" PFA) stream sucking the air either from the chamber exhaust or from the supply air at the flow rate of 12.5 L min^{-1} by an external pump. Additionally, a filter (material: PTFE) was used to discard particles that could potentially clog the capillary at the entrance of the Vocus. A calibration was performed before, during and after the campaign (in total 4 times) using a gas mixture standard (Apel-Riemer Environmental Inc), including acetaldehyde, methanol, ethanol, acetonitrile, acetone, acrylonitrile, isoprene, DMS, methyl vinyl ketone, methyl ethyl ketone, benzene, *m*-xylene, alpha-pinene, 1,2,4-trimethylbenzene, octamethylcyclotetrasiloxane (D4), decamethylcyclopentasiloxane (D5), 1,2,4-trichlorobenzene, and beta-caryophyllene. 6-MHO and 4-OPA were calibrated with individual standard gas bottles. For data processing, we used Tofware (version 3.2.5; Tofwerk AG and Aerodyne Research, Inc.) in the Igor Pro 7.08 environment (WaveMetrics, OR, USA). In order to identify if a VOC is related to human emissions, only masses having an increase higher than 3 times the standard deviation of that mass during the empty chamber period were further considered for chemical formula assignment. In terms of quantification of VOCs not presented in the gas standard, the mixing ratios were calculated based on the experimentally derived transmission curve using either known rate coefficient of that compound reacting with protonated water in the literature or a fixed rate coefficient at 2.5×10^{-9} cm^3 $molecule^{-1}$ s^{-1}.

Section S2. Methodology for estimating particle growth rate

Ultrafine particle growth rate (GR) is usually obtained by the t_{50} method, as shown in Fig S1-1 extracted from Lehtipalo et al.[3] The procedure is described as follows: (1) in a particle formation event, find the maximum concentrations (C_{max}) of each size bin and then normalize particle concentrations by the C_{max} for each size bin; (2) find the 50% appearance times (t_{50}) of the particles, corresponding to the elapsed time when the concentration reaches 50% of the C_{max}; (3) obtain particle GR by linear fitting between particle sizes and their corresponding t_{50}, after which the slope of the fitting stands for GR for a given size range.

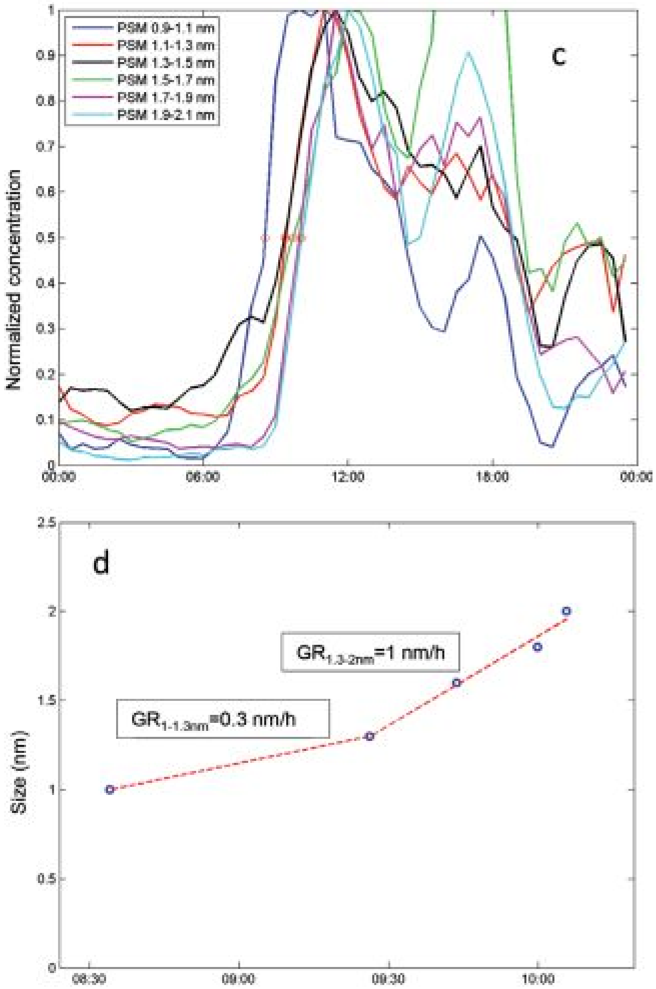

Fig S-I. A nucleation event day (31 Mar. 2011) at SMEAR II in Hyytiälä. (c) Normalized concentration in each size bin (maximum concentration in each bin set to 1); the 50% appearance times of the particles are marked with red circles. (d) The GRs determined with the appearance time method $GR_{sr,50}$ for two separate size classes 1–1.3 nm and 1.3–2 nm. Extracted from Fig. 7 in Lehtipalo et al.[3]

However, when the ultrafine particle concentrations are not high and have considerable fluctuations during a formation event, such as in our study, the t_{50} method may bring uncertainties in determining the maximum concentration for normalization and the 50% appearance time. It is expected that in a particle formation event, the time-series plot of larger size particles would have a similar shape to the smaller size particles, but with a time lag (indicating particle growth). Inspired by this feature we propose to use the cross-fitting method to estimate GR. The procedure is demonstrated in Fig S1-2 and described as follows: (1) apply time-series correlation between particle concentrations of the lower-bound size and larger sizes with adjustable time lags; (2) record the time lag where the highest coefficient is found for each size; (3) obtain particle GR by linear fitting between particle sizes and their corresponding time lags, in which the slope represents particle GR.

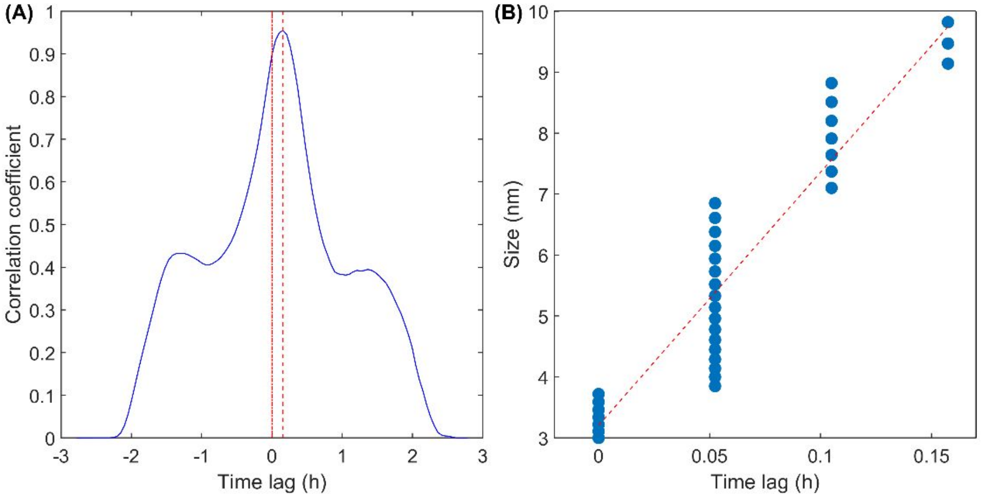

Fig S-II. An example of estimating particle growth rate using the cross-fitting method in one mixing-fan-off experiment with 3.0 h^{-1} air change rate. (A) Time-series correlation coefficient between 10 nm and 3 nm particle concentrations with adjustable time lags. The highest coefficient is marked with a dashed line, and the corresponding time lag indicates the delayed formation of 10 nm particles relative to 3 nm; (B) linear fitting between particle sizes and their corresponding time lags, in which the slope represents particle growth rate in the size range of 3-10 nm.

We compared the results obtained from the t_{50} method and the cross-fitting method. In the most obvious particle formation event in mixing-fan-off experiment with 3.0 h^{-1} air change rate, the results of particle GR within 3-10 nm were similar: 42.8 nm/h from the t_{50} method and 41.5 nm/h

from the cross-fitting method. It indicates the accuracy of the proposed method. On the other hand, the cross-fitting method worked for other experiments with much less particle formation, showing the robustness of this method. Therefore, this study reported particle GR obtained by the cross-fitting method, shown in Table 1.

Reference

(1) Lindinger, W.; Hansel, A.; Jordan, A. On-Line Monitoring of Volatile Organic Compounds at Pptv Levels by Means of Proton-Transfer-Reaction Mass Spectrometry (PTR-MS) Medical Applications, Food Control and Environmental Research. *Int J Mass Spectrom Ion Process* **1998**, *173* (3), 191–241. https://doi.org/10.1016/S0168-1176(97)00281-4.

(2) Krechmer, J.; Lopez-Hilfiker, F.; Koss, A.; Hutterli, M.; Stoermer, C.; Deming, B.; Kimmel, J.; Warneke, C.; Holzinger, R.; Jayne, J.; Worsnop, D.; Fuhrer, K.; Gonin, M.; de Gouw, J. Evaluation of a New Reagent-Ion Source and Focusing Ion–Molecule Reactor for Use in Proton-Transfer-Reaction Mass Spectrometry. *Anal Chem* **2018**, *90* (20), 12011–12018. https://doi.org/10.1021/acs.analchem.8b02641.

(3) Lehtipalo, K.; Leppä, J.; Kontkanen, J.; Kangasluoma, J.; Franchin, A.; Wimnner, D.; Schobesberger, S.; Junninen, H.; Petäjä, T.; Sipilä, M. Methods for Determining Particle Size Distribution and Growth Rates between 1 and 3 Nm Using the Particle Size Magnifier. *Boreal Environment Research* **2014**, *19 (suppl. B)*, 215–236.

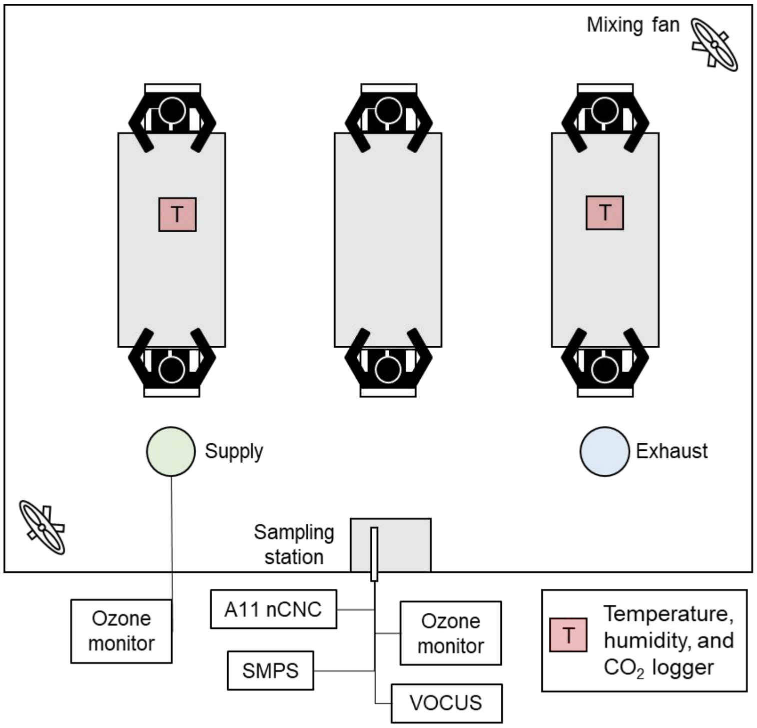

Fig. S1.
Schematic layout of the climate chamber with sampling locations for the experiments. The air was supplied via the supply diffuser and exhausted through a single outlet in the ceiling. Six participants were seated at three tables during the experiments. A11 nCNC, SMPS, and VOCUS were the instruments to measure real-time 1-3 nm nanocluster aerosols (NCAs), 3-55 nm ultrafine particles, and volatile organic compounds (VOCs), respectively.

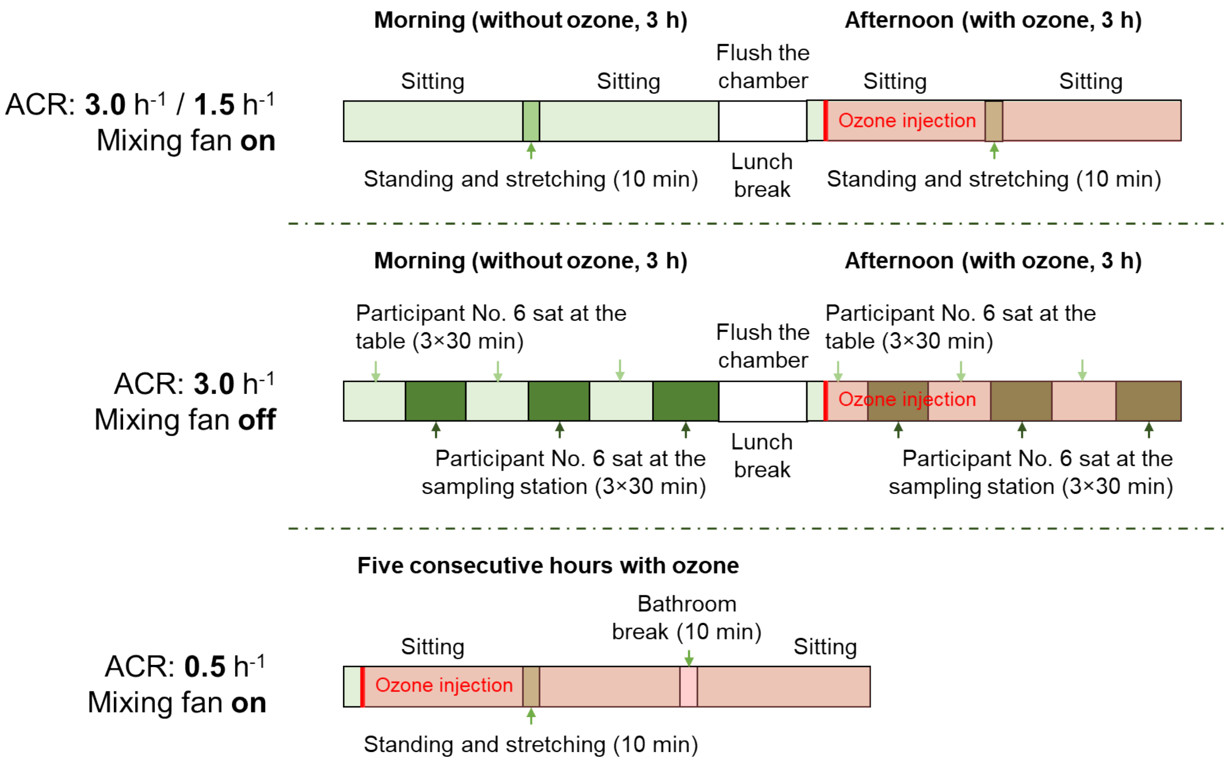

Fig. S2.

Illustration of experiment procedures in the scenarios of different air change rate (ACR) and mixing fan status. All experiments were performed with the same group of six participants. In the experiment with mixing fans off, one participant (No. 6) moved between the table and the sampling station, in order to investigate the potential difference in ultrafine particle levels between the bulk air and the peri-human microenvironment. In sessions with ozone, ozone was injected 10 min after the participants entered the chamber, targeting a steady-state level of 24-30 ppb inside the occupied chamber. The chamber conditions were set the night before each experiment to ensure that they had reached steady state at the beginning of the experiment. The chamber door was closed during the entire experiment. After the volunteers exited the chamber, the door was kept closed for 30 min and the decay of particle concentrations was measured.

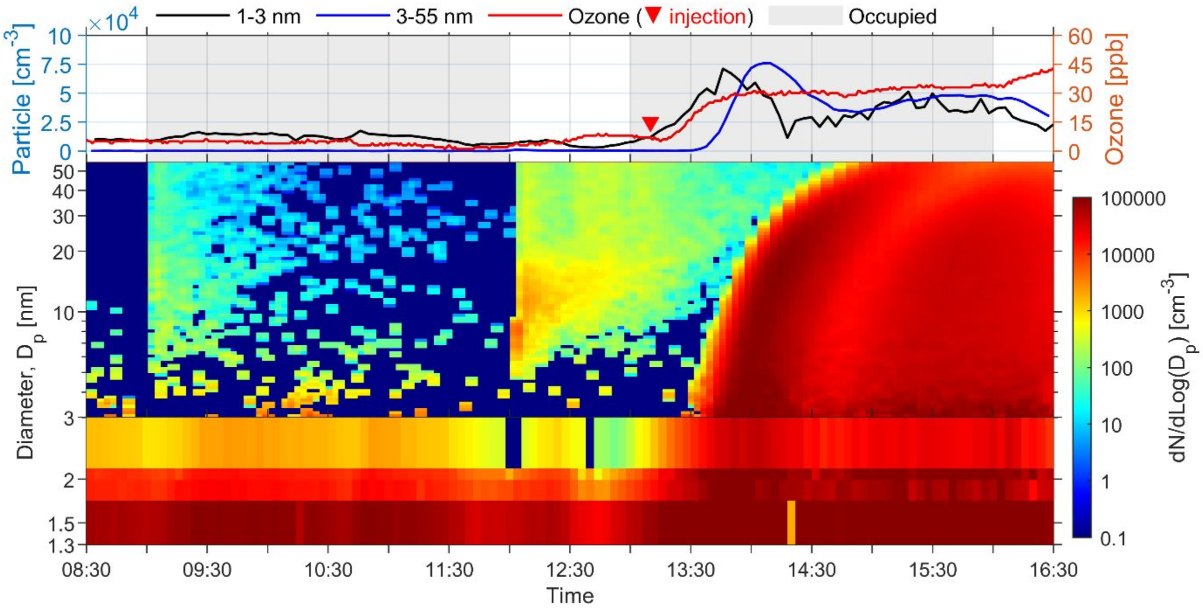

Fig. S3.

Time series of ultrafine particle concentrations and size distributions at 3.0 h^{-1} air change rate with mixing fans off. 1-3 nm particles were measured by A11 nCNC system and the diameter was activation size, whereas >3 nm particles were measured by SMPS and the diameter was mobility size. Shaded area in the top chart indicates the time when the chamber was occupied; and the upside-down triangle represents the moment when ozone was injected into the chamber.

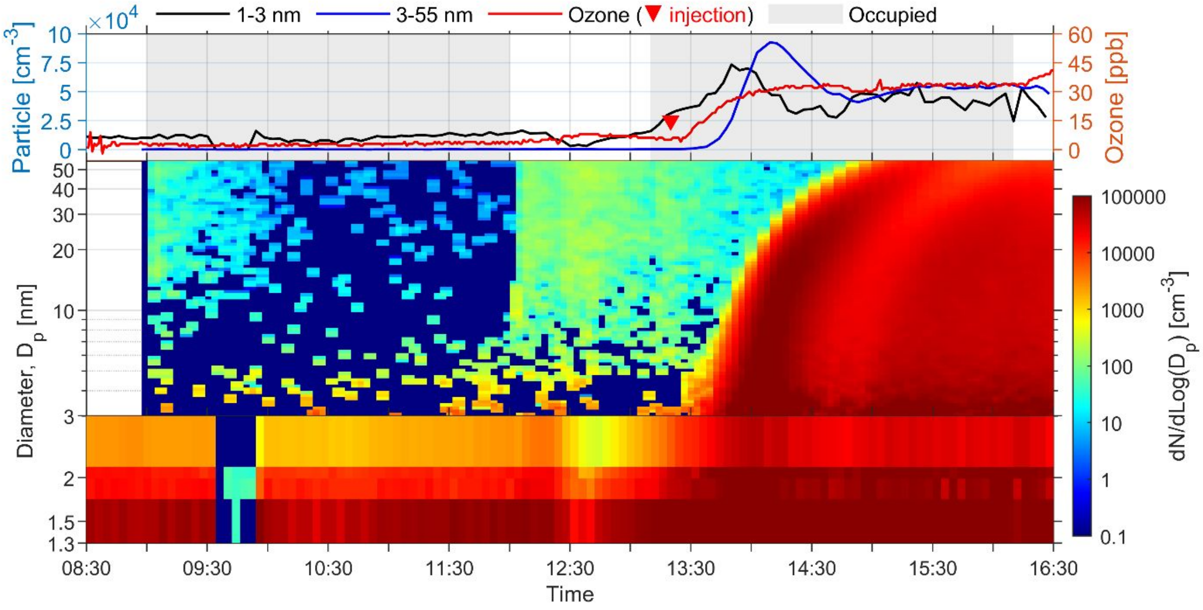

Fig. S4.

Time series of ultrafine particle concentrations and size distributions at 3.0 h^{-1} air change rate with mixing fans off (replicate). 1-3 nm particles were measured by A11 nCNC system and

the diameter was activation size, whereas >3 nm particles were measured by SMPS and the diameter was mobility size. Shaded area in the top chart indicates the time when the chamber was occupied; and the upside-down triangle represents the moment when ozone was injected into the chamber. Note that in the top chart, the presented 1-3 nm particle concentration was divided by 20.

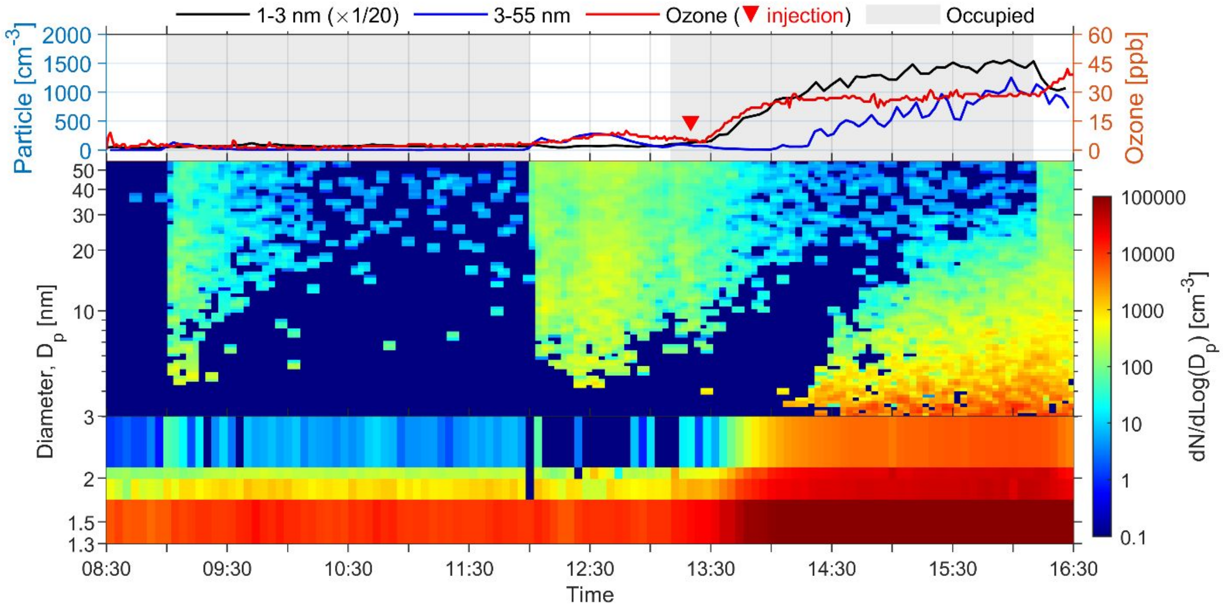

Fig. S5.

Time series of ultrafine particle concentrations and size distributions at 3.0 h^{-1} air change rate with mixing fans on. 1-3 nm particles were measured by A11 nCNC system and the diameter was activation size, whereas >3 nm particles were measured by SMPS and the diameter was mobility size. Shaded area in the top chart indicates the time when the chamber was occupied; and the upside-down triangle represents the moment when ozone was injected into the chamber. Note that in the top chart, the presented 1-3 nm particle concentration was divided by 20.

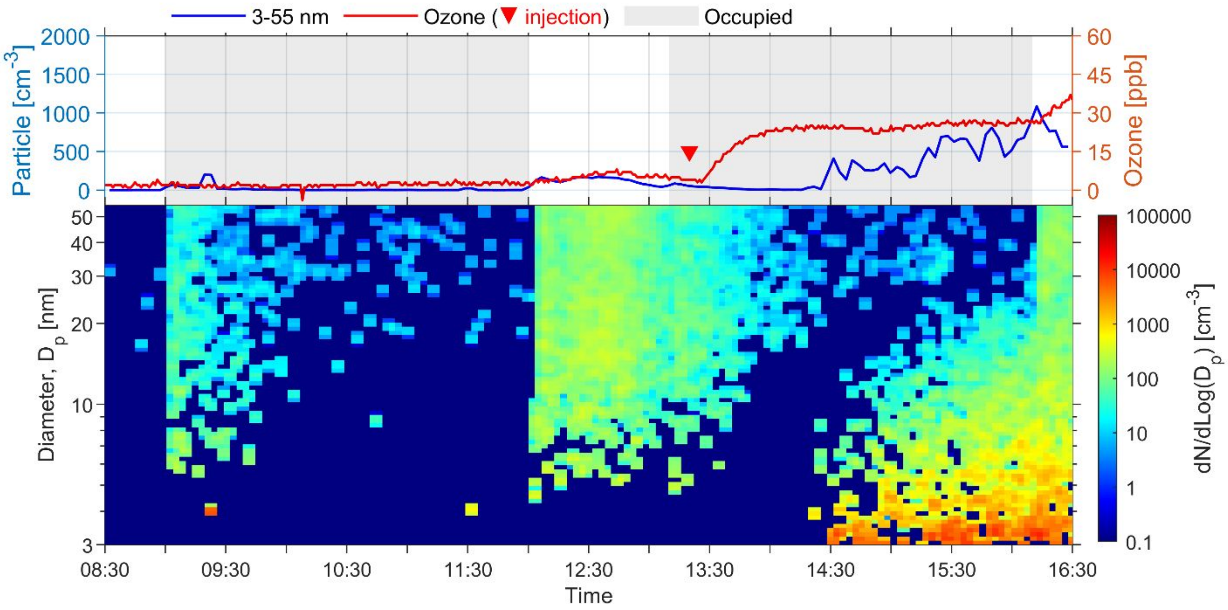

Fig. S6.

Time series of ultrafine particle concentrations and size distributions at 3.0 h^{-1} air change rate with mixing fans on (replicate). A11 nCNC system was unable to measure 1-3 nm particles in this experiment, whereas >3 nm particles were measured by SMPS and the diameter was mobility size. Shaded area in the top chart indicates the time when the chamber was occupied; and the upside-down triangle represents the moment when ozone was injected into the chamber.

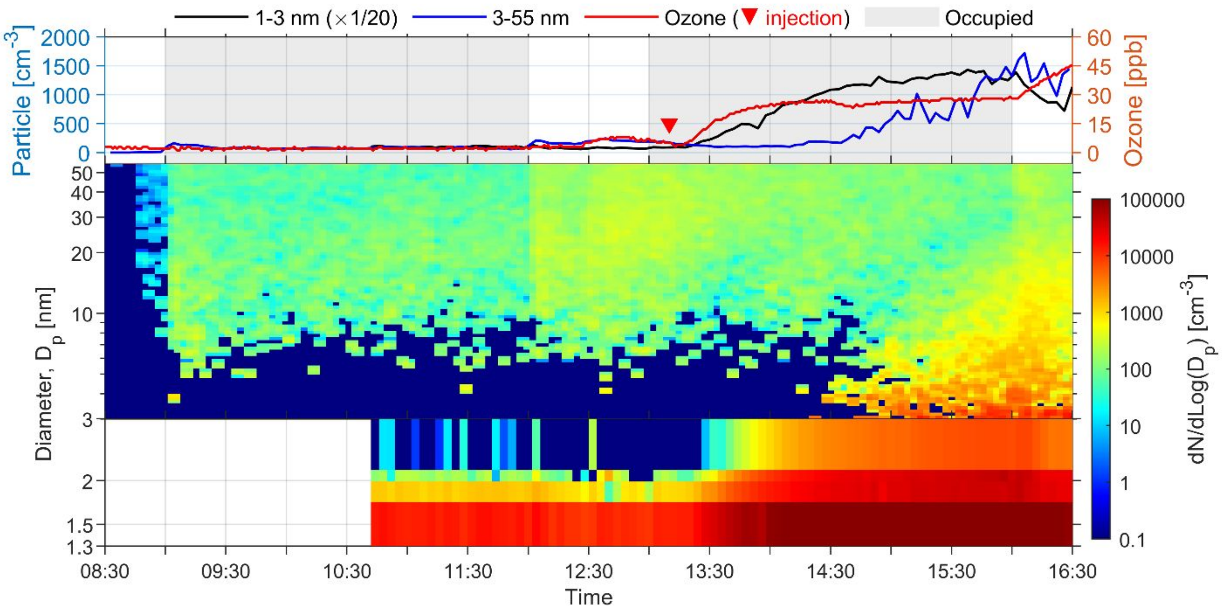

Fig. S7.

Time series of ultrafine particle concentrations and size distributions at 1.5 h^{-1} air change rate with mixing fans on. 1-3 nm particles were measured by A11 nCNC system and the diameter was activation size, whereas >3 nm particles were measured by SMPS and the diameter was mobility size. Missing data of 1-3 nm particles in the morning session was due to a data storage issue of the instrument. Shaded area in the top chart indicates the time when the chamber was occupied; and the upside-down triangle represents the moment when ozone was injected into the chamber. Note that in the top chart, the presented 1-3 nm particle concentration was divided by 20.

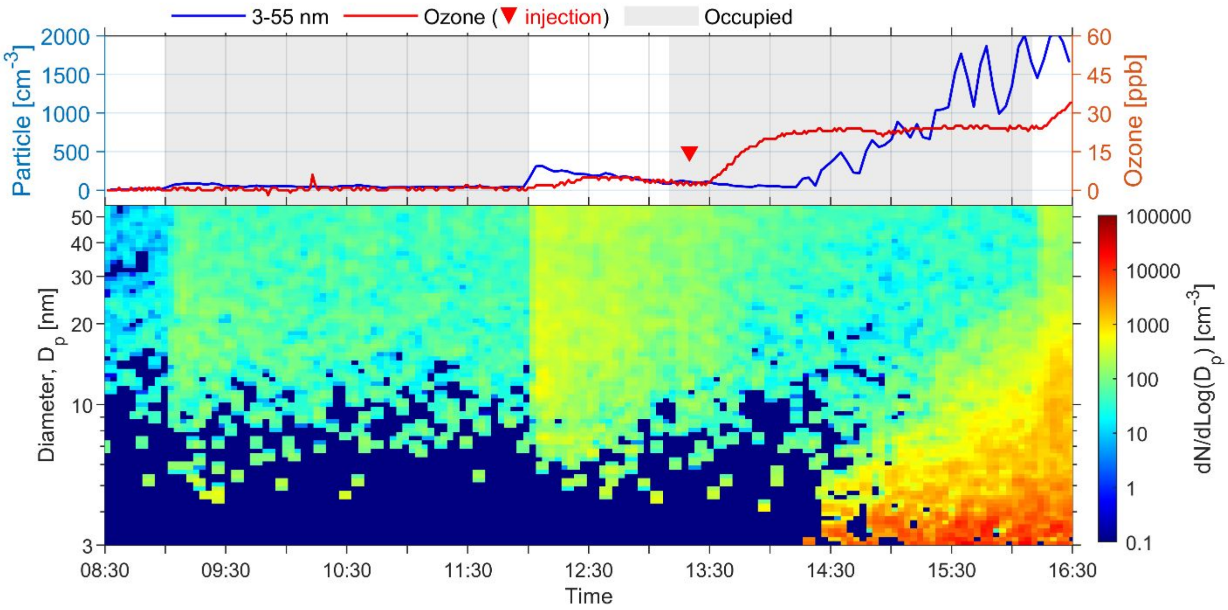

Fig. S8.

Time series of ultrafine particle concentrations and size distributions at 1.5 h^{-1} air change rate with mixing fans on (replicate). A11 nCNC system was unable to measure 1-3 nm particles in this experiment, whereas >3 nm particles were measured by SMPS and the diameter was mobility size. Shaded area in the top chart indicates the time when the chamber was occupied; and the upside-down triangle represents the moment when ozone was injected into the chamber.

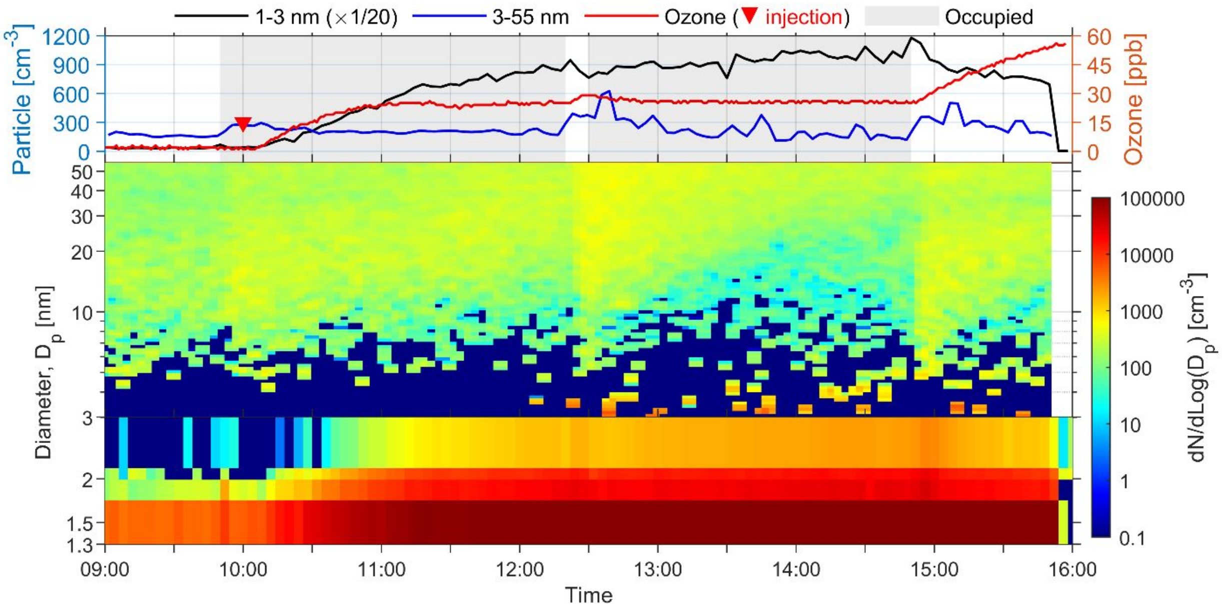

Fig. S9.

Time series of ultrafine particle concentrations and size distributions at 0.5 h^{-1} air change rate with mixing fans on (replicate). 1-3 nm particles were measured by A11 nCNC system and the diameter was activation size, whereas >3 nm particles were measured by SMPS and the diameter was mobility size. Shaded area in the top chart indicates the time when the chamber was occupied; and the upside-down triangle represents the moment when ozone was injected into the chamber. There was a 10-min bathroom break at 12:20. Note that in the top chart, the presented 1-3 nm particle concentration was divided by 20.

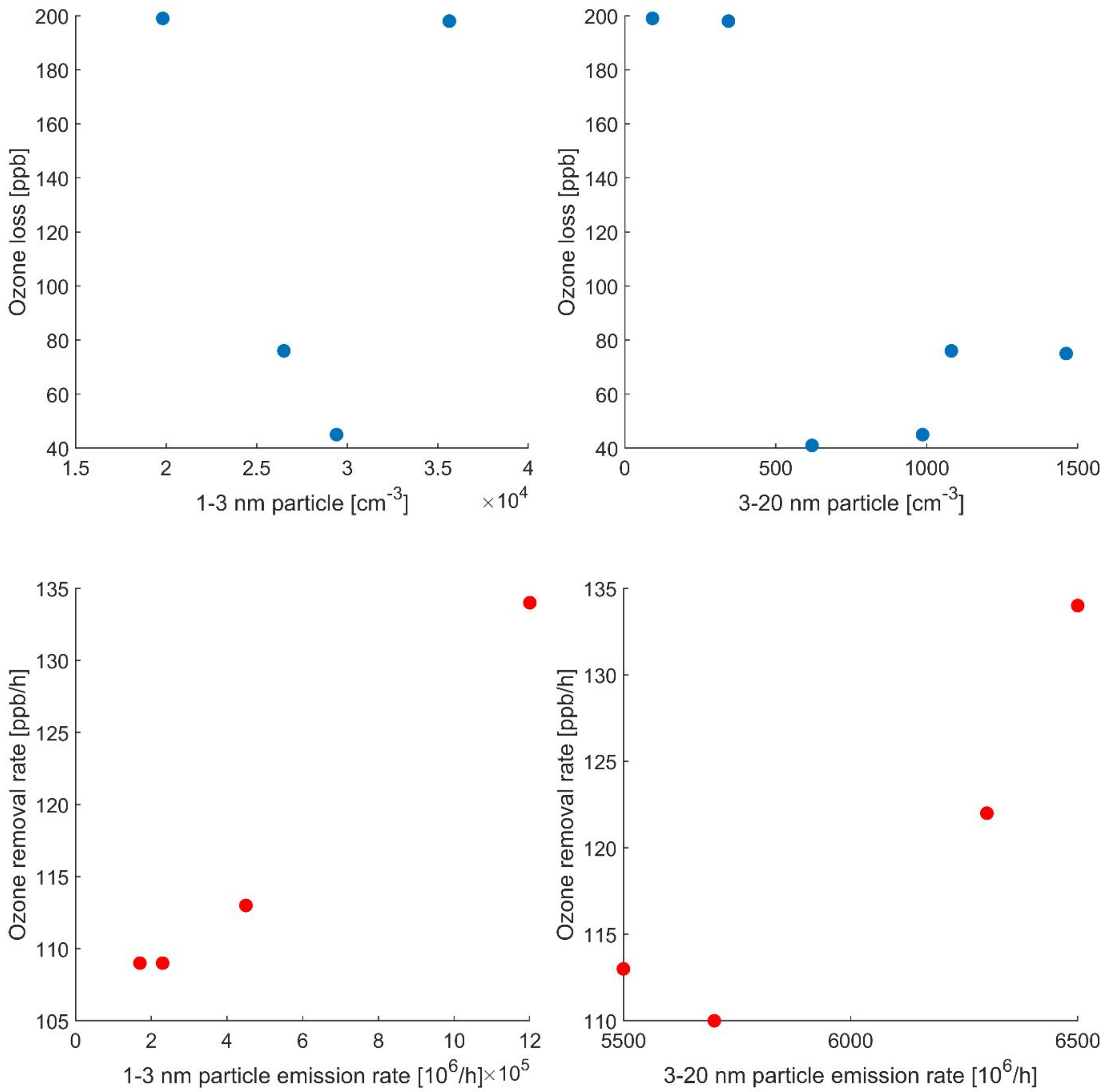

Fig. S10.

Correlation between quasi-steady-state concentrations of particles and ozone loss (upper) and correlation between particle emission rates and ozone removal rates (ozone loss times air change rate, lower) across all the experiments with mixing fans on.

Table S1.
Physiological data of participants in the experiments

Participant No.	Sex	Age	Height (cm)	Weight (kg)	BMI (kg/m^2)
1	Female	29	168	70	24.8
2	Male	21	185	63	18.4
3	Female	22	155	45	18.7
4	Female	21	168	75	26.6
5	Female	28	160	48	18.8
6	Male	29	165	67	24.6
